# Piloting home telepresence to support social connectedness among older adults in remote Irish communities

**DOI:** 10.3389/fpubh.2026.1796124

**Published:** 2026-06-05

**Authors:** Hemendra Worlikar, Leona Ryan, Ian McCabe, Jack Pinder, Jennifer Doran, Noreen Lineen-Curtis, Jane Walsh, Derek O'Keeffe

**Affiliations:** 1 Health Innovation Via Engineering (HIVE) Lab, School of Medicine, University of Galway, Galway, Ireland; 2 CÚRAM Research Ireland Centre for Medical Devices, Galway, Ireland; 3 School of Psychology, University of Galway, Galway, Ireland

**Keywords:** digital health, implementation science, older adults, social support, telepresence

## Abstract

**Background:**

Social support and social connectedness are key components of social health in later life, with reduced support linked to loneliness, social isolation, and adverse health outcomes. These challenges are intensified in remote and offshore communities, where geographic barriers limit social interaction and service access. In Ireland, nearly one in three adults over 50 experience emotional loneliness. While ICT-based interventions show promise, evidence for scalable, context-appropriate solutions remains limited. This study examines the feasibility and acceptability of a user-centred home telepresence approach in remote Irish settings.

**Methods:**

This nested case study, reported using the CASET framework, piloted a home telepresence intervention with older adults living alone in remote Irish communities. Following baseline surveys on expectations and preferences, participants engaged in scheduled video interactions. Post-intervention interviews assessed usability and general experience.

**Results:**

The telepresence intervention was readily adopted by older adults, demonstrating high usability and accessibility. However, tensions emerged between automation and user control, alongside evolving privacy concerns. Engagement was strongest when supporting shared activities beyond conversation. Participant feedback highlighted the need for greater flexibility, improved controls, and enhanced on boarding to support sustained use.

**Discussion:**

This case study demonstrates the feasibility of home telepresence as a rapid-deployment approach to support social connection among older adults in remote settings. Findings highlight the importance of usability, autonomy, privacy, and relational engagement for acceptability. Implementation Mapping supported structured, iterative development, informing future research, practice, and policy to enable sustainable, context-sensitive scale-up in rural and island communities.

## 1 Introduction

Social support and social connectedness are central components of social health in later life, capturing the availability of emotional, practical, and advisory support and the subjective sense of belonging, closeness, and being valued within meaningful relationships (1–3). These elements are particularly important for older adults, as weaker social support, reduced relationship quality, loneliness, and social isolation are associated with adverse health outcomes, including cardiovascular disease, stroke, cognitive decline, and poorer mental health (4, 5).

Social health encompasses an individual’s sense of social support and the degree to which their social needs are met. Composite measures of social health can identify individuals at risk, enabling targeted preventative interventions to bolster their social wellbeing (1). Understanding the significant impact of social wellbeing support across demographics, particularly among older adults, is urgently needed, considering ecological factors like community resources and access to services. This understanding must inform public expenditure to support older adults’ autonomy and address their specific social health needs (6). The use of an ecological framework to identify and assess risk factors for social isolation across four levels: individual, relationship, community, and societal while reviewing various interventions designed to reduce or prevent social isolation in later life, including one-to-one, group, service-based, technology-based, neighbourhood, and structural approaches. Notably, befriending and engaging group interventions have shown positive effects, while the growing use of social technology among older adults presents a promising avenue for combating isolation (7).

Social connectedness (SC) reflects the psychological bond people feel in relationships and toward others generally, and Hare-Duke et al. outlined this bond through five dimensions: closeness, shared identity, valuing relationships, social involvement, and feeling cared for and accepted (Hare-Duke et al., 2019) (2). SC is more associated with the quality of relationships than the number of interactions. It helps reduce loneliness and counters social isolation by fostering meaningful ties and support. Strong connections provide resources—emotional, practical, or financial—that can be drawn on when needed, showing why good relationships matter for wellbeing. These connections not only boost health and happiness but also help buffer stress, with their impact depending on the type and quality of the relationship (3). Social connection stands an essential human need, yet increasing numbers of people around the world report feeling emotionally disconnected. In Ireland, nearly one in three adults over 50 experience emotional loneliness, while in the USA, the number of people living alone has risen sharply over recent decades—trends that mirror similar or even higher rates of social isolation in both high- and low-income countries (8–10). Social isolation among the older adult often results from reduced social interactions due to retirement, physical or cognitive decline, bereavement, and living alone or in institutions. Information and communication technology (ICT) offers promise for overcoming these barriers, with studies showing positive effects from interventions such as videoconferencing, online chat, and even virtual pet companions (11).

The aim of this research is to examine the feasibility and acceptability of long-duration daily home telepresence for supporting social connectedness among older adults living in remote communities, with a particular emphasis on rural Irish contexts. This study is positioned as a feasibility and implementation-oriented investigation, focusing on the collaborative co-development of a user-centred and contextually appropriate telepresence methodology and protocol—defined as an automated video and audio connection between two devices—through direct engagement with older adults and their family members.

The overarching goal is to generate evidence-based insights that can inform the design of future interventions and policies tailored to the specific needs of individuals in geographically isolated settings, thereby contributing to improved quality of life and overall wellbeing. Subsequent phases of this work will build on the feasibility insights generated in the present study by incorporating validated measures of loneliness and social connectedness, alongside objective engagement metrics such as frequency and duration of use, to support more rigorous outcome monitoring and evaluation.

## 2 Methodology

This study was reported in accordance with the Case Study Evaluation Template (CASET; Goffin et al., 2019; Supplementary Material 1) (12). Ethical approval was granted for this study by the University of Galway Research Ethics Board (Ref: C. A. 3,178). The study was conducted in line with the Declaration of Helsinki, and written informed consent was collected from participants prior to taking part in the research. Participation was voluntary, participants were not compensated for their inclusion and, no participants dropped out of the study.

### 2.1 Setting

The study is nested within the Home Health Project, an international first initiative to address the challenges of delivering healthcare to Ireland’s most isolated communities (13, 14). The project focuses on Clare Island, constituting a population of 165 inhabitants. The project aimed to assess a digital health infrastructure to advance timely access to care for islanders; empower patients and, alleviate strain on an overburdened healthcare system. This is achieved through the use of remote monitoring devices to collect longitudinal data. This data then informs clinical decision-making supported by artificial intelligence, facilitating a proactive and tailored approach to the management of chronic conditions such as hypertension (14).

### 2.2 Design

This brief report outlines a nested case study that piloted the potential of a telepresence device to address social isolation among older adults in remote island communities. This is an established approach to investigate a phenomenon within its real life context, particularly where interactions between the participants, the phenomenon, and its context are not yet fully understood (15, 16). In accordance with the CASET template, the case boundaries were defined by the existing Clare Island Home Health Project cohort, specifically focusing on the interaction between isolated island residents and their mainland-based social supports. The primary unit of analysis was the dyad, rather than the individual, allowing for an examination of the relational communication flow facilitated by the innovation.

The methodology’s inherent flexibility facilitated the use of multiple data collection methods to generate insights into the phenomenon being explored (16). The methodology followed an iterative design where-in data collection and implementation were mutually informative. The process initiated with a qualitative survey to establish baseline expectations and preferences regarding telepresence interactions, call structures and technical support requirements. These survey insights directly informed the subsequent configuration of the communication protocol and the established schedule for the video-call sessions. Following the trial period, the cycle concluded with individual exit interviews to assess user experience and system adaptability. This iterative feedback loop ensured that the telepresence protocol was grounded in participant needs, providing a practical framework to inform future iterations of the device protocol for scalability.

### 2.3 Technology—Home telepresence setup

**Figure 1:**
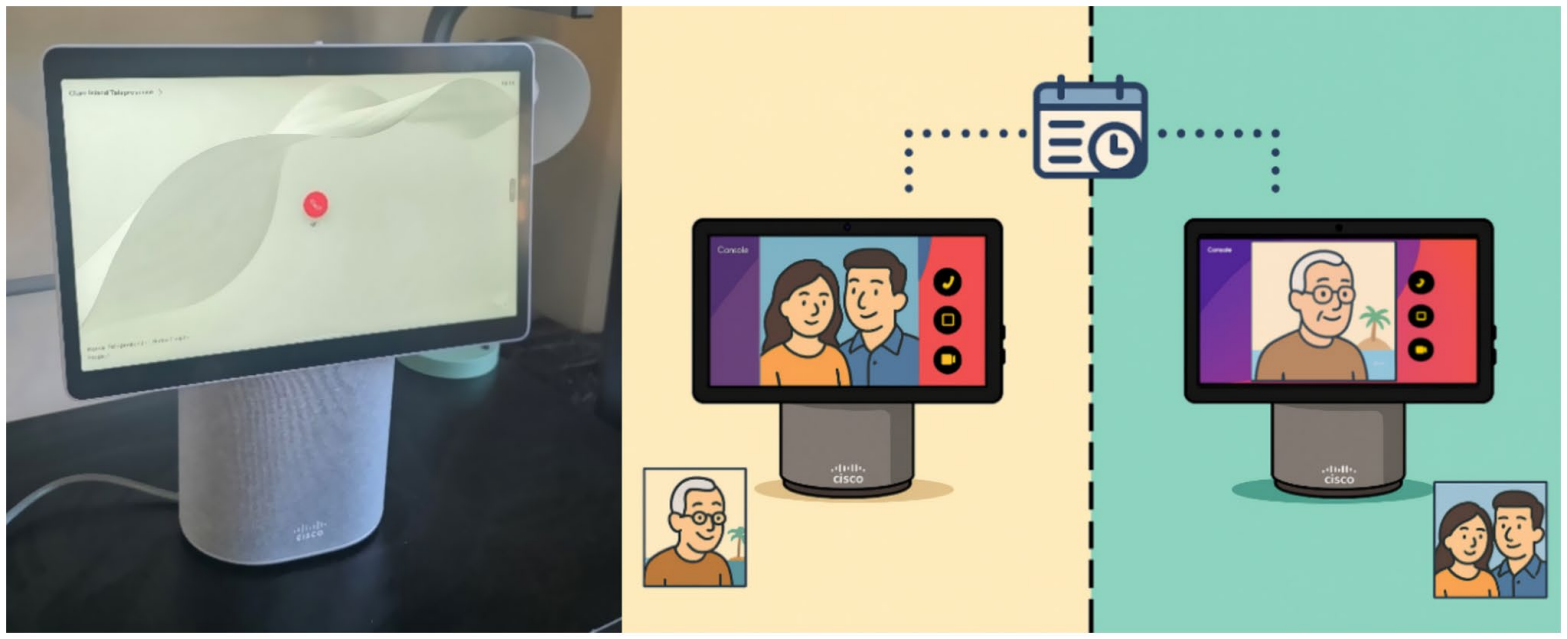
Cisco desk mini (left) home telepresence setup illustrating scheduled call workflow (right).

This study utilized two Cisco Desk Mini devices as presented in Figure 1, to support remote video communication between participants and their corresponding family member or friend. Each device integrates a high-resolution display, camera, speaker, and onboard computing into a compact, portable form factor, making it well-suited for deployment in diverse settings.

The devices run on Cisco’s proprietary RoomOS, which provides a standardized and intuitive user interface across endpoints. RoomOS enabled the automation of call functions, allowing the system to initiate and answer video calls based on an agreed pre-set schedule completed with the pre-survey. This feature minimized the need for participant interaction with the device and ensured consistent engagement throughout the study. Built-in WebEx functionality supported high-quality, real-time video calls, leveraging enterprise-grade security protocols. The setup was configured to meet strict privacy standards, with no local storage, video capture, or recording enabled. These safeguards ensured compliance with ethical requirements related to participant confidentiality and data protection. Overall, the configuration offered a secure, user-friendly, and reliable communication channel, enabling consistent remote interaction while maintaining a low technical burden on participants.

### 2.4 Procedure

The telepresence device was operated independently by each participant within their own residence. No researcher or third-party operator was present during the trial sessions. The older adult (OA) participants on Clare Island and the family member (FM) participants on the mainland each managed their respective hardware end-points. As the FM was located on the mainland, they were not physically present in the OA home to assist with or mediate the use of the device.

Interaction was direct and symmetrical. The system was configured to facilitate one-to-one communication between the two households without the need for remote operation by the family member. This separation of roles ensured that the OA engaged with the hardware independently, and the findings reflect their own ability to operate the technology without external support or technical facilitation.

### 2.5 Participant selection

Participants were recruited through purposive sampling from the Clare Island Home Health Project. Identification of candidates was supported by the project’s Patient and Public (PPI) collaborator (JP), the island General Practitioner (GP) and the resident island public health nurse. This selection process targeted individuals whose living situations and social requirements aligned with the research objectives. The final sample consisted of four participants (*N* = 4), configured into two dyads.

Each dyad comprised an older adult (OA) resident living alone on Clare Island and a corresponding family member (FM) residing on the mainland. The recruitment of adult children as telepresence partners was contextually motivated as the OA participants lived alone, their mainland-based children represented their primary source of informal social support and the most frequent interlocutors. In this study, both the OA and the FM were primary end-users of the telepresence technology. The trial evaluated a symmetrical communication link between two private residences, where both participants engaged independently with the hardware to maintain familial bonds and mitigate geographical isolation.

The sample composition included an older adult female (>65 years) and her adult son (<40 years); an older adult female (>80 years) and her adult daughter (>50 years). Eligibility for participation was contingent on two factors: the ability to access to high-speed broadband and the completion of the Home Health Project’s baseline health assessment. This assessment served as a functional screen to ensure participants possessed the cognitive and physical capacity for independent operation of the innovation. Due to the small population of Clare Island (165 inhabitants), the specific health data are not disclosed to protect participants right to confidentiality and prevent participant re-identification. This measure ensures strict compliance with General Data Protection Regulation (GDPR) standards and the ethical protocols governing this study.

### 2.6 Data collection

The qualitative survey was administered by JP and IM to all participants to inform the initial configuration of the telepresence device. These surveys were completed individually by both the OA participants and their FM to capture distinct participants’ preferences for call scheduling; device placement, engagement activities, perceived technical comfort levels and initial privacy concerns. This information was used to align the device set-up and call schedule with the needs and preferences indicated by the individual participants’ responses. Where preferences differed within a dyad, configurations were established jointly in collaboration with the research team.

The frequency and timing of the telepresence sessions were determined by the participants’ preferences as identified in the baseline survey. These scheduled interactions formed the basis of the trial, with the number of calls and their duration varying according to the needs of each dyad. On average, sessions for fixed automated scheduled calls lasted for 7,560 min collectively, with a total of 56 calls between the two dyads completed over the course of the study, over 2 weeks. Conversational content was not recorded to ensure participant privacy and to maintain the naturalistic setting of the home environment.

Following the trial, semi-structured interviews were conducted with participants and their corresponding family members to explore their experiences’ using the device. Interviews were held in-person and on-line via Zoom video-conferencing, interviews lasted approximately 20 min. Field notes were recorded after each interview to contextualise the researcher’s initial insights. The interviews were digitally recorded with the participants consent, transcribed verbatim, and transferred to NVivo software for qualitative data management and analysis.

### 2.7 Data analysis

**Figure 2:**
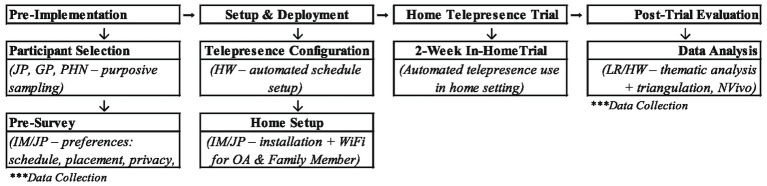
Home telepresence study flowchart.

Data were analysed using thematic analysis, guided by the six-step approach outlined by Braun and Clarke (17). This method was chosen for its flexibility and capacity for multi-layered data analysis, allowing us to generate a nuanced understanding of the participant experiences. The data from the survey and the qualitative interviews were triangulated to inform a comprehensive cross case comparison of participants’ perceptions before and after engaging with the telepresence device (15). The analysis was an iterative process illustrated in Figure 2 below. One author (LR) familiarised themselves with the data through repeated reading of the survey responses, interview transcripts and field notes to contextualise the data and to identify meaningful patterns in the data. Inductive coding was conducted at both the semantic and latent levels, with codes iteratively refined and organised into meaningful clusters. To ensure methodological rigor, two researchers (LR, HW) engaged in reflexive structured discussions at each stage of the analysis (18). This practice facilitated the consolidation of codes into thematic patterns, generating candidate themes and subthemes. To ensure congruency and coherence, the research team reviewed these themes at both the individual level and across the entire dataset. This collaborative process ensured the interpretations aligned with the research aims, ultimately resulting in the refinement and finalisation of themes and subthemes.

## 3 Findings

### 3.1 Usability and accessibility: a low barrier to entry

The fundamental design of the telepresence device established a low barrier to entry for both the OA and FM participants, regardless of their prior experience with technology. The perceived straightforward set-up and high-quality visual experience reportedly allowed users to quickly adopt the technology. This intuitive design was particularly evident for the OA participants, one of whom self-reported initially as being “*very uncomfortable with tech*” or having “*poor eyesight*,” yet managed to use the device effectively in their home without needing additional support: “*No, except for my eyesight. But the quality was good. What adjustments can we [sic] make to better accommodate your [sic] accessibility needs?[…] New eyes?!”* (OA2). The FM participants similarly reported that the system required no technical oversight, facilitating a symmetrical ease of use. This suggests that the core interface and hardware were suited to the target demographic on the island and provided a reliable connection for the FMs on the mainland. Overall, the simplicity of the device supported initial user confidence and allowed it to function as a stable medium for connection: “*It was great. The picture and sound were very good*” (OA2); “*The quality of image[…] and being able to see my mother clearly*” (FM2).

The trial demonstrated a low barrier to entry, as participants could engage with the technology without significant technical training. However, once the device was integrated into the home, several design flaws emerged that impacted the overall user experience. The most frequent request from both OA and FM participants was for an immediate and reliable “*hang-up button,”* indicating a fundamental user need for manual control over the connection: “*Generally easy to use but [..]pressing ‘Call’ on Qdial did not stop the call*” (OA1).

Similarly, persistent issues with being “*able to turn down the volume*” (OA2) and a desire for a “*screen angle needs to be adjustable*” (OA1) were frequently mentioned, particularly within the OA population. These requests highlight the need for more customisable hardware features that allow the users to adapt the device to their immediate environment rather than relying on a one-size-fits-all approach.

Regarding the set-up protocol, the mainland-based FM participants, who lacked the same direct access to the research team as the island cohort identified a need for proactive resources such as “*video tutorials.”* Conversely, the OA participants requested a more detailed “*walkthrough*” and suggested that the trial period should be extended. They reportedly felt a longer duration was necessary to move beyond the initial learning curve and build the trust and comfort required for the technology to feel integrated into their daily lives.

### 3.2 Control and flexibility

The convenience of an automated, fixed schedule was initially chosen by all participants in the baseline survey. However, the interview data revealed a perceived tension between system automation and user autonomy. Both the OA and FM participants initially agreed to predetermined call times but the rigid nature of these sessions reportedly transformed the opportunity for connection into an obligation that created a sense of unease and in one household became a source of stress.

This finding was more evident among the FM participants who had to manage the schedule around the presence of other family members and the unpredictable movement of a multi-person household. In contrast to the OA participants who lived alone on the island, the FM participants found that the lack of flexibility afforded by the predetermined scheduling made it difficult to rearrange sessions when “*life happened*.” For one mainland family, this lack of autonomy over the schedule created enough pressure that they reported being “*happy not to have it in our house*” by the end of the trial: “It’s a nice idea to have the automated telepresence sessions, however, by the end of the trial period we were happy not to have it in our house.” (FM2). This finding suggests that while the initial configuration was mutually agreed upon, the innovation required a higher degree of user-initiated control. Future iterations should prioritise facilitating user-initiated calls and customisable schedules to ensure the innovation empowers participants to fit the device into their lives, rather than the other way around.

### 3.3 Privacy

The concept of privacy was found to be a dynamic perception that shifted with the participant’s experience of engaging with the device, rather than a static concept that could be fully captured in the initial survey. For the OA participants, privacy issues were often related to the physical placement of the hardware within the home. One OA initially reported having “*no privacy concerns*”, yet this was challenged during the trail by the realisation that the camera angle “*captured a view of the bathroom*” (OA1). In contrast, the FM participants on the mainland experienced privacy concerns related to the social environment of a family household. An important observation was made by one FM in relation to who may be in the home and the nature of the pre-defined schedule. It was perceived that the pre-defined schedule lacked the flexibility to account for the presence of others, FM participants both requested a way to turn off the device manually, citing concerns that unexpected visitors or younger family members could overhear their private conversations: “*Yes, you became quite self-conscious about what you were saying when just walking around your house. Particularly given that other people may have been in my mother’s house (e.g. guests) without our knowledge. It happened a couple of times that there were visitors within earshot but not within view of the camera*” (FM1).

This suggests that the concept of privacy cannot be addressed as a checklist item in an initial set-up survey. The protocol for future rollouts may benefit from moving beyond an initial checklist to including a proactive onboarding process that explicitly addresses evolving privacy concerns. Embedding a system for continuous check-ins would ensure that both OA and FM participants can adjust device parameters in response to their lived experience. This iterative approach would support user trust by acknowledging that privacy needs may change once the technology is integrated into the home environment.

### 3.4 Engagement: beyond conversation to shared activities

When using the device, dyadic conversation was reportedly the primary method of connection, however, the device’s ability to facilitate shared activities with mainland family members was a key finding. The most positive and memorable feedback came from the OA participants, for whom the interaction shifted form standard conversation to active engagement, such as “*reading stories with grandchildren*”. These moments were highlighted as the most meaningful aspects of the trial, suggesting that the technology’s potential lies in its ability to host shared experiences rather than just video conferencing: “*Felt like they [FM1] were in the house*” (OA1).

The technology was noted for its value in a solitary context, with one participant highlighting that it is “*good for a person on their own*” (OA2). This is particularly significant given the island setting, where the device serves as a bridge for those living in isolation. This desire for deeper engagement led to specific technical suggestions from the mainland. One FM participant explicitly requested a “*screen sharing component*” to facilitate these interactions. This indicates that as the dyads moved past the initial learning curve, they were actively seeking ways to enrich their connection. Future iterations should therefore shift the design philosophy from a simple communication tool to a platform that supports collaborative activities, directly supporting the social connectedness that both OA and FM cohorts identified as a priority.

“*It was a pity it didn’t work out for longer (length of trail)” (OA2); “It a good use [sic] of modern technology, would participate again!*” (OA1).

## 4 Translating findings into practice: future directions

### 4.1 Implementation mapping

Implementation Mapping will be applied to guide the next iteration of the study protocol, grounding its development in both the empirical data generated in this study and established implementation theory. Building on the framework proposed by Fernández et al. (2019), providing a structured, theory-driven approach for specifying multi-level implementation strategies (19). In this study, the Implementation Mapping framework is primarily intended to support researchers and service-level facilitators (e.g., healthcare providers, community coordinators) in planning, refining, and operationalising telepresence interventions within real-world settings.

**Figure 3:**
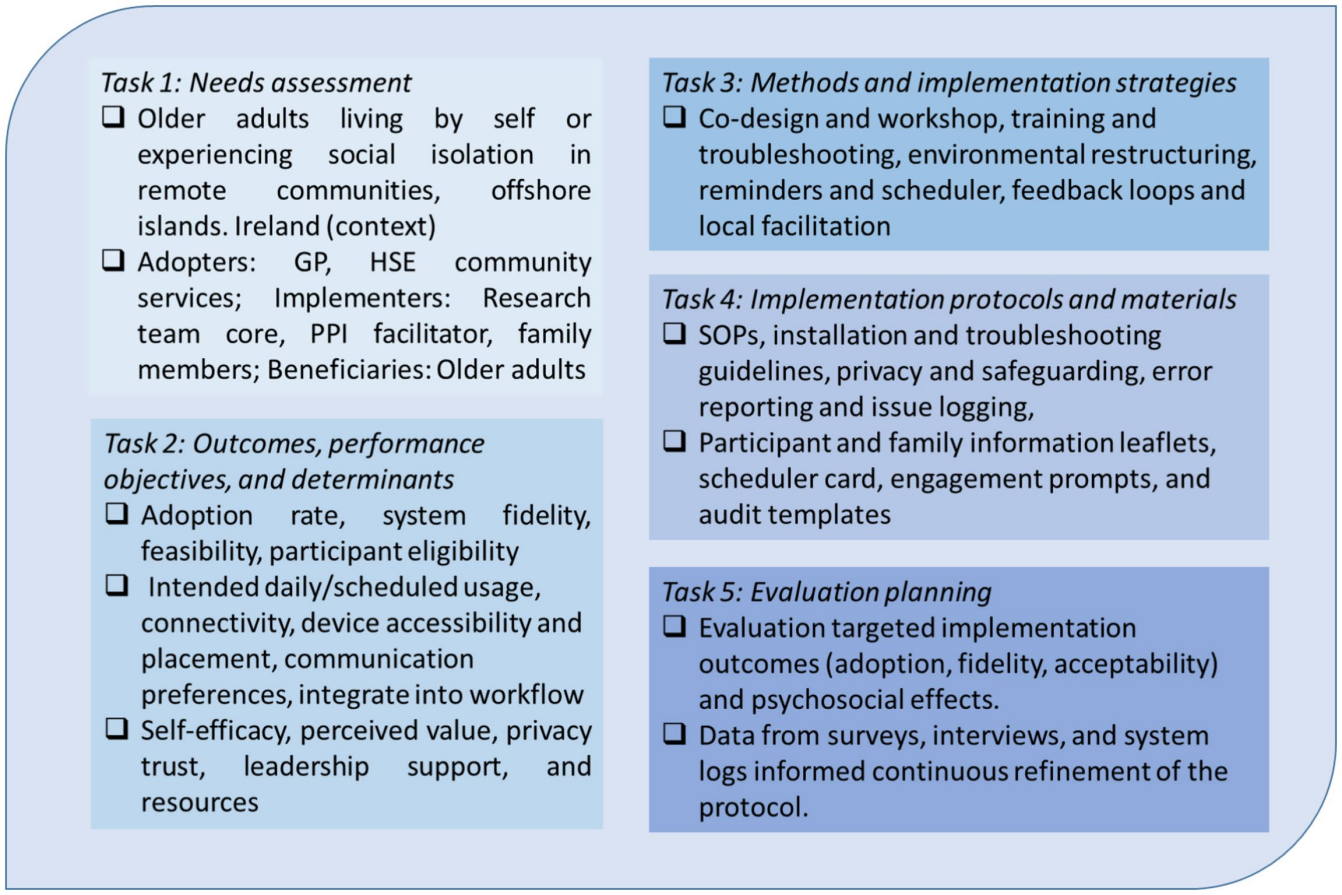
Implementation mapping of the home telepresence intervention.

In practice, the framework supports stakeholder coordination by clearly delineating the roles of key actors, including adopters (older adults and family members), implementers (researchers and facilitators), and supporting organisations (health and community services). It provides a structured process for translating empirical findings into actionable implementation strategies, including protocol development, training requirements, system configuration, and environmental adaptations tailored to the remote island context. In the next phase, the home telepresence intervention will be refined using the above framework as outlined in Figure 3 to further address the identified needs of older adults experiencing social isolation in geographically isolated settings.

The findings from surveys, interviews, and system-log data will inform the specification of priority outcomes, performance objectives, and key determinants, which will guide the selection and refinement of co-designed strategies, training supports, environmental adaptations, and protocol materials. The framework also functions as an implementation guide for scaling and replication, enabling systematic planning of deployment across similar remote communities. This approach will support systematic evaluation of adoption, fidelity, and acceptability in the subsequent iteration, with built-in mechanisms for ongoing refinement. Overall, the application of Implementation Mapping will enable a targeted, evidence-informed progression of the telepresence protocol, supporting iterative improvement and sustained uptake across implementation stages.

### 4.2 Technical refinements for the next iteration

**Figure 4:**
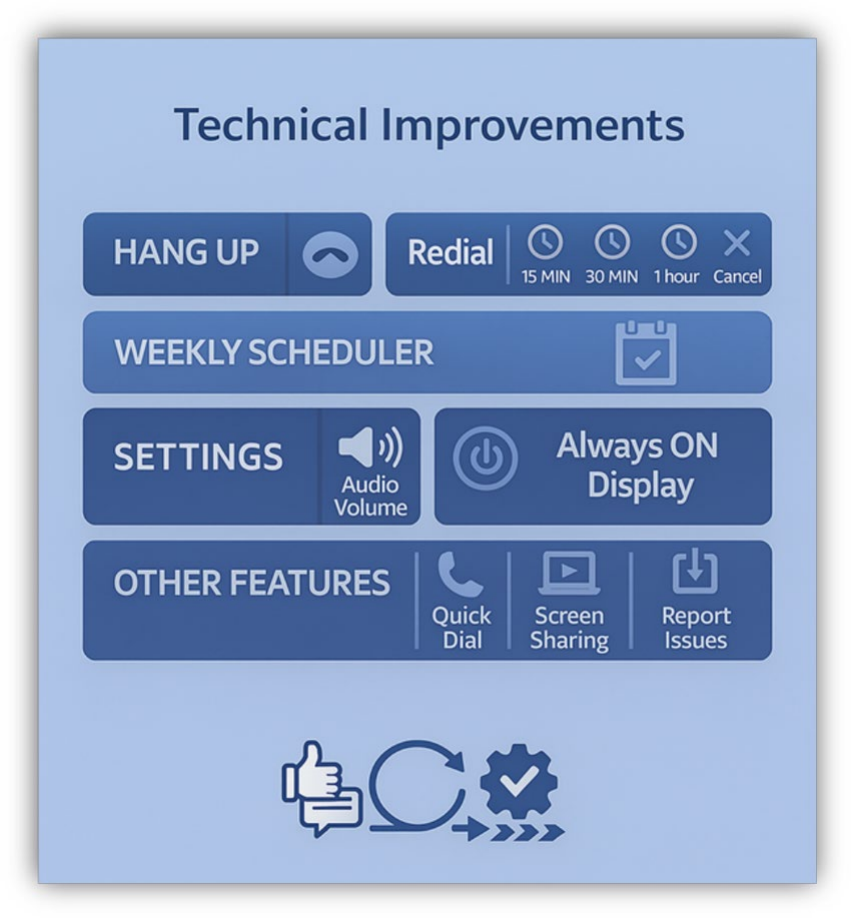
Feedback-driven technical improvements.

Building on empirical findings and participant feedback from the present study, these insights will inform the next iteration of the telepresence protocol. While automated call scheduling and redial functionality supported call continuity during dedicated sessions, this logic overrode the manual hang-up feature, limiting users’ ability to independently terminate calls. Participants reported this as unsatisfactory, highlighting the need for greater user control. In response, the next iteration will refine call-termination functionality and optimize automated redial options (15-min, 30-min, 1-h, and cancel) to better align with user expectations and autonomy (Figure 4).

Feedback from participants and family members also underscored the need for greater flexibility in managing call times. Planned enhancements include a more intuitive weekly scheduler, a home-screen audio settings icon, an “always-on” mode to support persistent device linkage, and a quick-dial function for on-demand calling. Additional refinements will include a screen-sharing guide and a streamlined “report issues” function linked to a dedicated email channel. Together, these refinements underscore the importance of embedding a closed-loop feedback mechanism within the system’s development cycle, enabling ongoing process optimisation and continuous improvement. By systematically integrating user insights with technical evaluation, the platform can evolve responsively to better meet the needs of older adults and their supporting networks.

## 5 Discussion

### 5.1 Feasibility and user experience—Home telepresence system

This nested case study explored the feasibility and acceptability of a home telepresence system for older adults living in a remote Irish island community. The study focused on the system usability and its experience in everyday settings. The telepresence device demonstrated a low barrier to entry, with participants able to use the system effectively regardless of prior technical experience or minor visual limitations. The device interface and hardware supported ease of use for older adults on the island and provided a reliable connection for family members on the mainland, contributing to initial user confidence and consistent use. Participants indicated that a longer trial period was needed to move beyond the initial learning phase and develop the trust required for integration into daily routines. The system was perceived as particularly useful for individuals living alone, supporting consistent interaction and connection within the island location. The findings highlight that telepresence can support perceived social connectedness in geographically isolated settings, while also identifying challenges related to user autonomy, privacy, and integration into daily routines. These findings align with existing literature on digital social-support interventions, which report mixed outcomes and emphasise the role of context, with the remote island setting in this study illustrating how distance from services and family networks shapes technology adoption and sustained use (3, 6). This contributes to current research by demonstrating how feasibility and acceptability are shaped by local conditions, rather than by technological capability alone.

Although participants initially selected an automated schedule for convenience, interview data identified a tension between system automation and user autonomy, with limited control over call timing and termination contributing to discomfort during use. Concerns arose regarding who may be present in the home during scheduled calls, with fixed scheduling lacking flexibility to account for others in the environment. Requests for a manual option to turn off the device reflected the need for greater control over privacy and use. Simplicity supports initial engagement, while flexibility in user control is required to sustain comfort, autonomy, and continued use in everyday contexts. Privacy concerns evolved during real-world use, particularly when the fixed call schedule risked exposing conversations to visitors or non-participating family members. These dynamic perceptions highlight the context-dependent nature of privacy in home-based interventions and indicate the need for proactive on boarding, flexible device placement, and periodic check-ins to support user trust and comfort. This reinforces the importance of treating privacy as an ongoing, adaptive process rather than a one-time configuration step during setup.

ICT-based methods to support social connection among older adults are well established, with prior studies signifying the potential of telepresence and participatory design interventions in this space (20, 21). The contribution of this study lies not in the technology itself, but in the application of a patient-centred, context-sensitive approach that responds to the social isolation needs and engagement patterns of older adults in remote island settings. This work provides feasibility and user-experience insights that inform the co-development of a telepresence protocol, supporting iterative refinement, implementation planning, and future outcome evaluation.

### 5.2 Engagement and dyadic interaction

A notable strength of the telepresence system was its support for shared activities, which participants described as particularly meaningful and engaging. These interactions suggest that telepresence can facilitate richer relational experiences beyond conversation, indicating potential pathways through which perceived social connectedness may be supported, rather than demonstrating direct psychosocial impact. User requests for features such as screen sharing further reflect a desire to deepen these shared experiences. This suggests that engagement was enhanced when interactions moved toward collaborative experiences, especially in a solitary island context. However, interpretations of relational or emotional outcomes remain limited. Notably, these findings highlight the importance of social affordances such as convenience, connectivity, and the transmission of social cues, which enable diverse and meaningful interactions and are theorised to support social connectedness and potentially mitigate loneliness (3).

### 5.3 Implementation and design implications

The Implementation Mapping approach supported a structured understanding of how a Home Telepresence system could be refined and implemented within this context. In this study, the framework functioned primarily as a planning and implementation tool for researchers and facilitators, rather than as an evaluative model of social outcomes. By linking programme activities to actor-specific objectives, determinants, and evaluation measures, the framework enabled clearer interpretation of usability, adoption, and implementation requirements, supporting the translation of qualitative insights into actionable protocol refinements.

Technical limitations, particularly the automated redial function overriding participants’ ability to end calls, reduced user control and highlighted the need for more flexible scheduling and reliable call termination features. User feedback also indicated demand for quick-dial options, clearer settings, and streamlined issue reporting, indicating that continuous, closed-loop refinement is essential for ensuring the system evolves to meet the needs of older adults. Together, these recommendations highlight the interdependence of technical design and implementation processes in shaping user experience. They also reinforce the need for iterative, user-centred development cycles that position older adults and their families as active contributors to system evolution. Direct interaction with the system revealed technical limitations and prompted shifts in user expectations, reflecting evidence that hands-on experience can reshape cognitive–affective responses to technology from baseline beliefs. When users and supporting actors perceive the system positively, their willingness to facilitate its intended deployment helps offset usability challenges, illustrating how system design, user experience, and stakeholder behaviours collectively influence the sustainability of digital interventions for older adults (22).

### 5.4 Limitations and future directions

The study is limited by its small sample, lack of pre–post psychosocial measures, and reliance on self-reported experiences. The absence of validated baseline and follow-up measures of loneliness and social connectedness limits the ability to draw conclusions about psychosocial impact. Short trial duration, context-specific conditions, and evolving technical issues further restrict generalisability. A further limitation is the lack of objective engagement metrics, such as call duration, disruptions, and user-initiated interactions thereby constraining precise assessment of engagement and fidelity, and perspectives of broader stakeholder groups were not captured, reducing insight into system-wide implementation factors. Designing policy to improve digital literacy among older adults is essential, as those most vulnerable to severe social isolation are often least able to access or navigate technology-based interventions. Enhancing access and awareness of such programmes is therefore critical for equitable implementation (7, 23). Information and Communication Technology can help older adults maintain connections across time and distance, supporting social inclusion when paired with tailored training that fosters confidence and positive attitudes (11). However, high attrition rates and mixed evidence for loneliness reduction indicate that such interventions may not suit all older adults. These findings indicate that public health policies could be strengthened by adopting more integrated, multi-component approaches to effectively address social isolation (24).

## 6 Conclusion

To conclude, this case study illustrates that home telepresence can serve as a feasible and acceptable, rapid-deployment approach to support social connection among older adults in remote communities, provided that its design and implementation carefully address usability, autonomy, privacy, and the relational qualities of interaction. Dyadic interaction experiences shaped participants’ overall perceptions of telepresence use, with shared activities emerging as particularly meaningful. The findings demonstrate the feasibility of integrating such systems into everyday home environments while highlighting contextual and emotional factors that influence acceptability. Extended use supports user confidence and demonstrates the value of telepresence in facilitating connection for individuals living alone in remote settings. The Implementation Mapping framework provided a structured approach for identifying actors, tasks, and determinants, supporting a coherent and iterative implementation process. Future work should incorporate validated measures of loneliness and social connectedness, alongside objective engagement metrics, to more rigorously evaluate outcomes. Next probable roll-out to additional island or rural regions will require continued technical refinement, personalised onboarding, flexible scheduling systems, and policy support to ensure sustainable, equitable adoption of telepresence-based social interventions.

## Data Availability

The raw data supporting the conclusions of this article will be made available by the authors, without undue reservation.
