# Supplementary material for: Piloting home telepresence to support social connectedness among older adults in remote Irish communities
Source: Front Public Health. 2026 Jun 5;14:1796124. doi: 10.3389/fpubh.2026.1796124 (PMC13278964; doi:10.3389/fpubh.2026.1796124)
Supplement: Supplementary file 1 [file Table_1.DOCX]

| **Table 1: Case Study Evaluation Template** | | |
| --- | --- | --- |
| **Evaluation Criteria** | **Explanation of Measure** | **Evidence** |
| Theoretical Foundation | Was a clear explanation given of why the case method was the most appropriate method to adopt? | Yes. The study is described as a nested case study designed to investigate a phenomenon within its real-life context, specifically where interactions between participants, the phenomenon, and the context (remote island life) are not yet fully understood. |
| Pilot Study | Was there a pilot study preceding the main study? | No. This study serves as the pilot phase for a larger planned intervention. As a pilot case study, its primary purpose is to test the feasibility of the telepresence protocol and use the generated empirical data to inform and refine the design, technical requirements, and implementation mapping for the subsequent main study. |
| Theoretical Sampling | Was an explanation provided of which cases were chosen and why? | Yes. Purposive sampling was used to select two dyads (*N*=4) consisting of older adults living alone on Clare Island and their mainland-based adult children. This island-mainland structure was chosen to evaluate a symmetrical communication link designed to mitigate geographical isolation. |
| Triangulation | Was the research based on multiple sources of data? | Yes. The study utilised data triangulation by combining baseline qualitative surveys, semi-structured exit interviews, and researcher field notes recorded throughout the trial. |
| Review and Validation of evidence | Was the evidence reviewed and validated by external parties? | Yes. The findings were validated through reflexive structured discussions between two researchers to ensure all themes were grounded in the empirical data. In addition, the thematic insights were reviewed and discussed with the project’s PPI collaborator and the extended research team. This process ensured that the interpretations were aligned with the lived realities of the island community and the broader objectives of the Home Health Project. |
| Transparency of data collection | Was it made clear how the data collection process was conducted? | Yes. The manuscript outlines a three-stage process: a baseline phase (qualitative surveys to establish initial preferences), an independent trial phase (active two-week device deployment in participant residences), and an evaluation phase (individual semi-structured exit interviews). |
| Inter-coder agreement | Were the data coded by multiple investigators? | Yes. To maintain methodological rigor, the data were initially coded by a one researcher who then engaged in reflexive structured discussions at each stage of the analysis with a second researcher. This collaborative process was used to challenge initial assumptions, consolidate codes, and refine the resulting themes, ensuring the findings were consistently grounded in the data. |
| Case presentation | Were findings and empirical evidence presented in a way that made it clear how the authors reached their conclusions? | Yes. Findings are organised into four themes and are supported by verbatim illustrative quotations from both Older Adult (OA) and Family Member (FM) participants. |
| Case interpretation | Did the case analysis move beyond description to conceptual ordering? | Yes. The analysis transitioned from descriptive reporting of participants feedback to a conceptual ordering of findings that directly informed the project’s next steps. These insights were translated into the Implementation Mapping framework, which delineates the specific roles of adopters and beneficiaries and defines the performance objectives for the next iteration of the trial. This grounding of the findings in the users' lived experiences on the island and the mainland within the framework provides a structured analytical foundation for the systematic refinement of the telepresence protocol and the technical design of the device for future rollout. |
| Reflecting on validity and reliability | Was there a discussion about the quality of the research? | Yes. The Discussion includes a clear and reflexive consideration of the study’s validity and reliability, with explicit acknowledgement of methodological strengths and limitations. The study is positioned as a feasibility and implementation-focused investigation, emphasising experiential and contextual findings and avoiding causal claims regarding reductions in loneliness or social isolation. Importantly, the limitations section critically highlights the absence of validated pre–post psychosocial measures of loneliness and social connectedness, noting that this constrains the ability to assess psychosocial impact. Acknowledging the small sample size and lack of objective engagement metrics strengthens the credibility of the findings and supports appropriate methodological rigour for an exploratory case study design. |
